# Supplementary material for: Extracting phylogenetic dimensions of coevolution reveals hidden functional signals
Source: Sci Rep. 2022 Jan 17;12:820. doi: 10.1038/s41598-021-04260-1 (PMC8764114; doi:10.1038/s41598-021-04260-1)
Supplement: Supplementary file 1 — Supplementary Information. [file 41598_2021_4260_MOESM1_ESM.docx]

**Supplementary Information for “Extracting phylogenetic dimensions of coevolution reveals hidden functional signals”**

Alexandre Colavin^1,*^, Esha Atolia^2,*^, Anne-Florence Bitbol^3,4,5^, Kerwyn Casey Huang^6,7,8,†^

^1^Biophysics Program, Stanford University School of Medicine, Stanford, CA 94305, USA

^2^Department of Chemical and Systems Biology, Stanford University School of Medicine, Stanford, CA 94305, USA

^3^Sorbonne Université, CNRS, Institut de Biologie Paris-Seine, Laboratoire Jean Perrin (UMR 8237), F-75005, Paris, France

^4^Institute of Bioengineering, School of Life Sciences, Ecole Polytechnique Fédérale de Lausanne (EPFL), CH-1015 Lausanne, Switzerland

^5^SIB Swiss Institute of Bioinformatics, CH-1015 Lausanne, Switzerland

^6^Department of Bioengineering, Stanford University, Stanford, CA 94305, USA

^7^Department of Microbiology and Immunology, Stanford University School of Medicine, Stanford, CA 94305, USA

^8^Chan Zuckerberg Biohub, San Francisco, CA 94158

^*^: Co-first authors.

^†^To whom correspondence should be addressed: [kchuang@stanford.edu](mailto:kchuang@stanford.edu)

**Supplementary Figures**


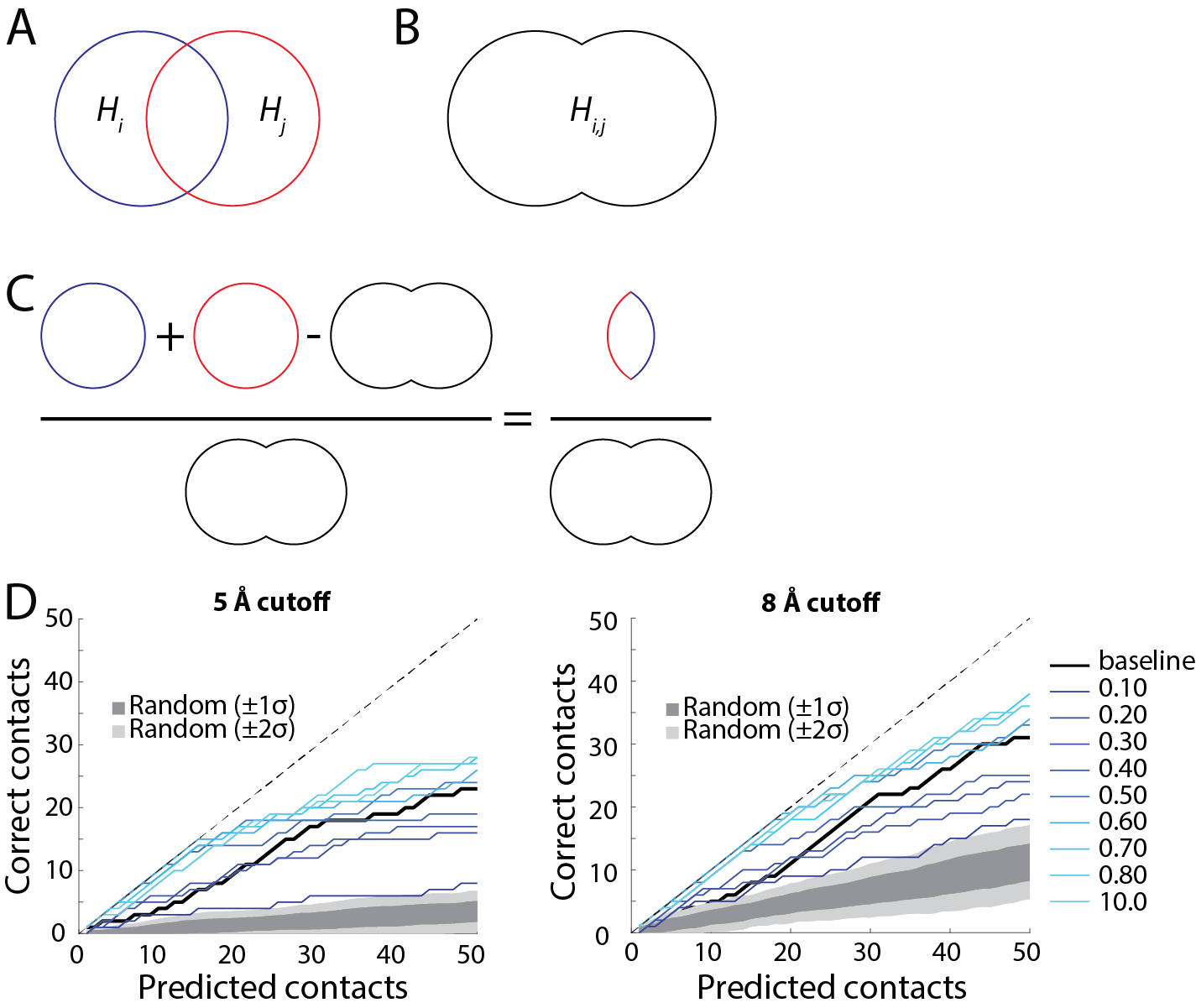


**Figure S1****: Pictorial representation of information-theoretic metric of residue-residue coupling, and comparison of structural-contact prediction at different cutoffs.**

1. Protein positions *i* and *j* that take on multiple amino-acid identities in an MSA have non-zero entropies, represented by the areas of *H_i_* and *H_j_*. The two circles are overlapping because knowing the identity of position *i* provides some information about the identity of position *j*.
2. The union of the space occupied by positions *i* and *j* is the joint entropy.
3. The normalized mutual information metric of coevolution (Eq. 1) represents the sum of the entropies in (A) minus the joint entropy in (B), normalized by the joint entropy in (B).
4. NC with particular cutoffs *d* improves the prediction of structural contacts in the protein KH-1 relative to DCA, applied to DI after correction with APC (black lines). Results were similar whether structural contacts were considered to be within 5 Å (left) or 8 Å (right) at closest approach. All residues within five positions on the polypeptide sequence were excluded from the analysis. Gray represents the predictions of the baseline NMI metric.

**Figure S2: Relationship between magnitude and significance of NC values.**

1. NC measurements by bootstrapping and analytical methods (Methods) are highly correlated.
2. The significance of NC, estimated by bootstrapping (Methods), is highly correlated with its magnitude, demonstrating that magnitude of NC signal is a surrogate for its significance.

**Figure S3: NC can improve structural true-positive contact prediction over DI + APC.**

NC delivers a net improvement in recovering structural information by DCA, which is stronger for breadth-sampled MSAs than depth-sampled MSAs, compared to baseline (in this case DI with APC). NC recovers breadth better than depth, in absolute terms highlighting the importance of having breath in an MSA. NC provides approximately the same fold-change in improvement across subsampling degrees.


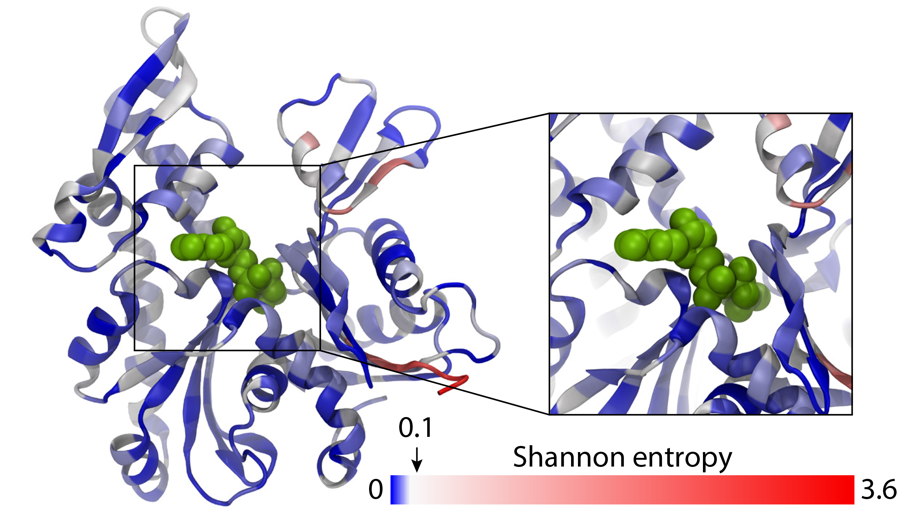


**Figure S4: The ATP binding pocket of MreB is composed of highly conserved residues.** Amino acids are colored by their Shannon entropy, with blue indicating values <0.1. The ATP molecule is shown in green.


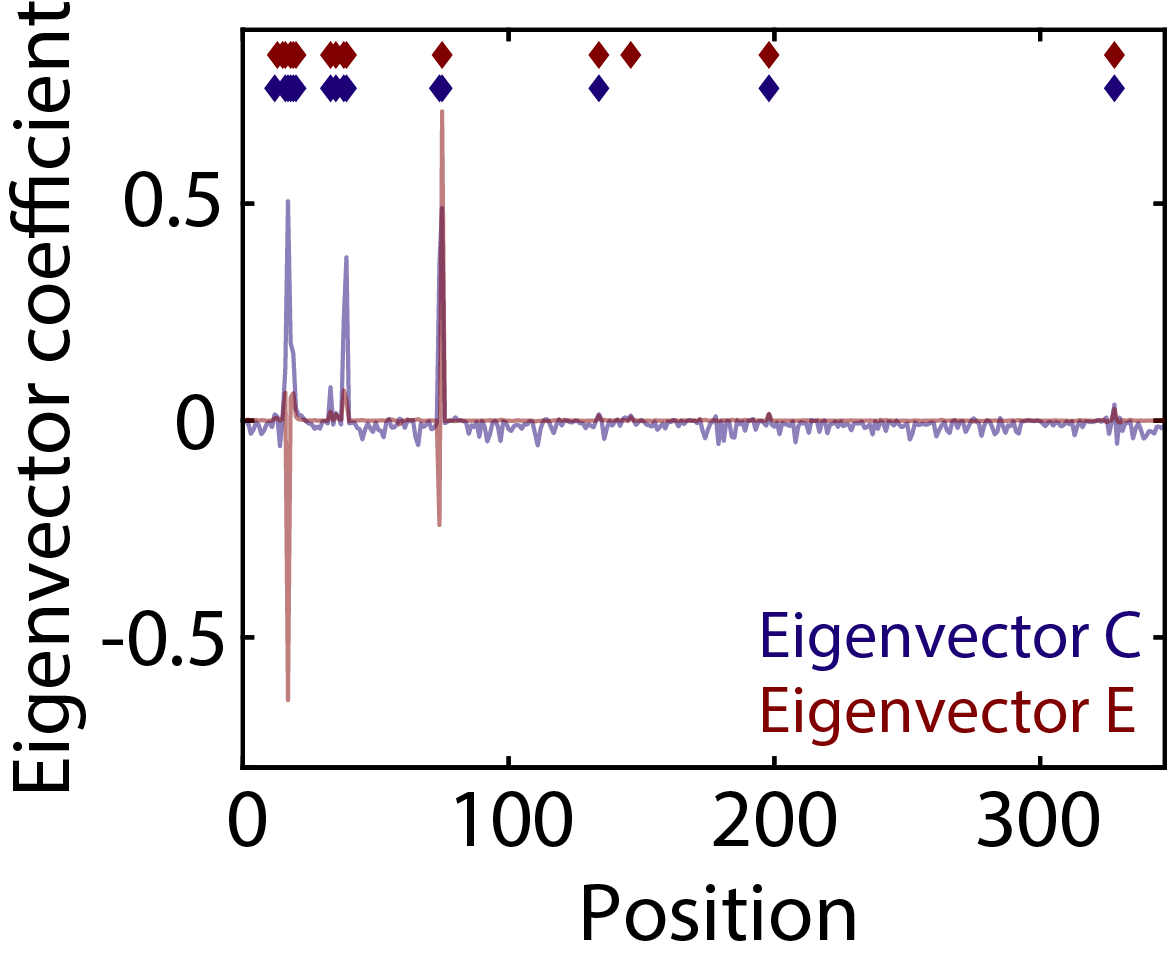


**Figure S5: Similar eigenvectors are merged to create meta-sectors.** The positive potions of eigenvectors C and E define sectors C and E, respectively. These eigenvectors are combined to create a meta-sector.

**Figure S6: The maximum relative enrichment for H-Ras sectors is similar to the enrichment for the protein overall.** The maximum enrichment profile for sectors A (A) and B (B) are similar to that of the overall protein (black) for both NC and baseline.


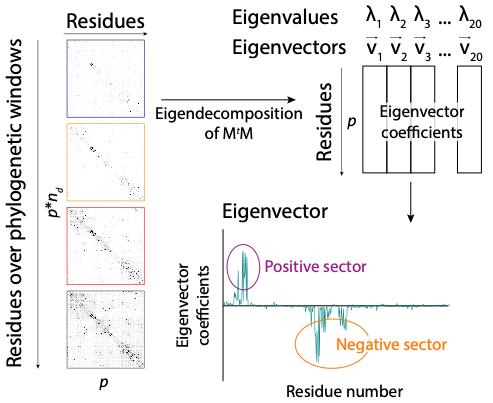


**Figure S7: Schematic showing sector determination.** The NC sectors are concatenated, eigendecomposition of M*^t^*M (where M is the supermatrix and M*^t^* is its transpose) is performed, and the eigenvectors associated with the highest eigenvalues are used to define positive and negative sectors.

**Supplementary Table**

| **Protein** | **Uniprot ID** | **NCBI accession number** | **PDB ID** |
| --- | --- | --- | --- |
| Cadherin | I3LUS1_PIG | - | 2O72 |
| Enolase | - | NP_417259 | 5OHG |
| FKBP-C | A0A0D2TZB3_GOSRA | - | 1R9H |
| G6PD | - | NP_000393 | 2BH9 |
| H-Ras | - | 1BKD_R | 1BKD |
| KH-1 | B4MJF9_DROWI | - | 1WVN |
| Kunitz | F6WEH0_ORNAN | - | 5PTI |
| Lectin-C | H0YSP5_TAEGU | - | 2IT6 |
| MAPK1 | - | NP_620407 | 6FN5 |
| MreB | - | NP_417717 | 2WUS, 1JCG, 4CZE |
| Ras | A0A024W598_PLAFA | - | 5P21 |
| RNase H | R9T5K3_9EURY | - | 1F21 |
| SH3 | A0A0K0FYI6_9BILA | - | 2HDA |
| Thioredoxin | H0ZH31_TAEGU | - | 1RQM |
| Trypsin | I3M2H8_ICTTR | - | 3TGI |

**Table S1: Reference sequences and PDB structures.**
